# Optimized Ultrasound-Assisted Extraction for Enhanced Recovery of Valuable Phenolic Compounds from Olive By-Products

**DOI:** 10.3390/antiox14080938

**Published:** 2025-07-30

**Authors:** Xavier Expósito-Almellón, Álvaro Munguía-Ubierna, Carmen Duque-Soto, Isabel Borrás-Linares, Rosa Quirantes-Piné, Jesús Lozano-Sánchez

**Affiliations:** 1 Department of Food Science and Nutrition, University of Granada, Campus Universitario s/n, 18071 Granada, Spain; expositox@ugr.es (X.E.-A.); carmenduque@ugr.es (C.D.-S.); 2 Department of Analytical Chemistry, Faculty of Sciences, University of Granada, Campus Fuentenueva s/n, 18071 Granada, Spain; alvaromun@ugr.es (Á.M.-U.); rquirantes@ugr.es (R.Q.-P.)

**Keywords:** olive pomace, olive leaves, phenolic compounds, ultrasound-assisted extraction, HPLC–ESI-QTOF-MS

## Abstract

The olive oil industry generates by-products like olive leaves and pomace, which are rich in bioactive compounds, especially polyphenols. This study applied a circular economy approach to valorize these residues using green ultrasound-assisted extraction (UAE) with GRAS solvents. Key parameters (solvent composition, ultrasound amplitude, and specific energy) were optimized via Response Surface Methodology (RSM) to enhance polyphenol recovery and yield. Ethanol concentration proved to be the most influential factor. Optimal conditions for olive pomace were 100% ethanol, 46 μm amplitude, and 25 J∙mL^−1^ specific energy, while olive leaves required 72% ethanol with similar ultrasound settings. Under these conditions, extracts were prepared and analyzed using HPLC-ESI-QTOF-MS and DPPH assays. The optimized UAE process achieved yields of 15–20% in less than 5 min and under mild conditions. Optimal extracts showed high oleuropein content (6 mg/g in leaves, 5 mg/g in pomace), lower hydroxytyrosol levels, and minimal oxidized derivatives, suggesting reduced degradation compared to conventional methods. These findings demonstrate UAE’s effectiveness in recovering valuable phenolics from olive by-products, supporting sustainable and efficient resource use.

## 1. Introduction

The olive oil industry is a key pillar of the Mediterranean region’s economy and culture, ranking among the most globally important agricultural sectors. Recognized for producing olive oil, a cornerstone of the Mediterranean diet, this industry has thrived for millennia. Countries such as Spain, Italy, Algeria, Turkey, Portugal, and Greece are leaders in olive cultivation and oil production [1]. Olive oil is highly valued, not only for its outstanding nutritional benefits and sensory attributes, but also for its cultural and economic significance [2]. However, despite these advantages, the production process generates substantial amounts of by-products, posing significant environmental and economic challenges [3].

The olive oil production process consists of several stages, including harvesting, washing, crushing, malaxing, and extraction. Two of the primary by-products generated during extraction using the two-phase centrifugation system are olive leaves and olive pomace. In Spain alone, more than 4 million tons of olive pomace and 0.2 million tons of olive leaves are produced annually [1]. The management of olive mill by-products, particularly olive pomace and olive leaves, results in measurable carbon emissions and financial costs. Life cycle assessments estimate that olive oil production generates approximately 1.6 kg of CO_2_ equivalents per liter of olive oil [4]. While specific data on the isolated contribution of waste management are lacking, the large volumes produced suggest that it accounts for a substantial share of these emissions. In addition, waste management costs in Spain typically range from €2 to €8 per ton [5], representing a significant net expense for olive mills. Therefore, implementing sustainable strategies to reduce greenhouse gas emissions and mitigate the economic burden associated with the management of olive pomace and olive leaves is essential.

In recent years, the concept of circular economy has gained traction as an effective approach to waste management in the food industry. Within this framework, researchers have increasingly turned their attention to the revalorization of by-products from olive oil production [6,7,8]. These materials hold considerable promise for sustainable applications, including their use in composting, energy generation, animal feed, and soil improvement [9]. Additionally, their high content of bioactive compounds, including phenolic compounds, provides opportunities for innovative uses in industries such as pharmaceuticals and cosmetics. With the growing global emphasis on sustainability, the olive oil industry is well-positioned to convert these by-products from waste into valuable resources, integrating economic efficiency with environmental responsibility [3].

Within the valuable bioactive compounds present in olive by-products, it is essential to highlight the primary phenolic compounds, particularly secoiridoids and phenolic alcohols. Among the secoiridoids, oleuropein is the most characteristic compound in olive-derived products. It primarily acts as an antioxidant, as its hydroxyl groups enable it to donate hydrogen atoms that prevent oxidative processes [10]. Further studies have shown that oleuropein also exhibits cardioprotective, neuroprotective, and anticancer properties [10,11]. The aglycone form of oleuropein also deserves attention, as it significantly contributes to the anti-inflammatory and antioxidant activities associated with various health benefits, including cardiovascular and neurological protection [12]. Additionally, oleacein is another relevant secoiridoid, recognized for its antioxidant and anti-inflammatory effects, as well as its beneficial role in cardiovascular health, particularly in conditions such as atherosclerosis [12,13]. On the other hand, within the group of phenolic alcohols, hydroxytyrosol stands out as a particularly relevant compound. This simple o-diphenol is formed through the hydrolysis of oleuropein during the ripening and storage of olives, and even more extensively during the olive oil production process, contributing to the oil’s chemical complexity and sensory diversity [14]. Its notable health benefits are largely attributed to its capacity to neutralize free radicals and reactive oxygen and nitrogen species, as well as its ability to enhance endogenous antioxidant defense mechanisms. As a result, hydroxytyrosol exhibits a broad range of bioactive properties, including antioxidant, anti-inflammatory, anti-aging, anticancer, and antimicrobial effects [15]. Additionally, it has been shown to reduce plasma cholesterol levels, further supporting its cardioprotective potential [16,17].

The revalorization of olive oil industry by-products requires the efficient extraction of specific bioactive compounds, which remains challenging due to their coexistence with numerous other constituents. Furthermore, the extraction process must not only ensure high efficiency, but also adhere to environmentally sustainable principles. In recent years, ultrasound-assisted extraction (UAE), particularly in combination with green solvents, has emerged as a promising strategy for the selective isolation of phenolic compounds from a wide range of plant matrices. UAE utilizes ultrasonic waves to generate cavitation (a phenomenon characterized by the formation, growth, and collapse of microbubbles), which facilitates the disruption of plant cell walls and enhances the release of intracellular compounds [18]. This eco-friendly technique has been successfully applied to the extraction of phenolic compounds from various olive-derived by-products, including olive pomace, using both water and natural deep eutectic solvents [19], as well as from olive leaves using ethanol–water mixtures [20].

While UAE offers several advantages (such as reduced solvent consumption, lower energy requirements, and increased extraction efficiency), its implementation at an industrial scale remains limited. This limitation stems primarily from the lack of standardized methodologies and the high variability in critical extraction parameters, including extraction time, temperature, and ultrasonic power. These parameters are often optimized under specific laboratory conditions that are not easily transferable between different ultrasound devices and rarely consider industrial scalability. In addition, many of the extraction protocols reported in the literature lead to the partial degradation of phenolic compounds, resulting in the formation of oxidized or hydrolyzed derivatives that compromise the functional quality of the extracts [18].

Despite the abundance of research on UAE, there is no clear consensus regarding which operational parameters most significantly influence extraction selectivity and efficiency, particularly when dealing with complex matrices such as olive pomace and leaves [21]. The novelty of the present study lies in the development of an optimized, reproducible, and industrially scalable UAE protocol for olive oil by-products (specifically olive leaves and olive pomace), with a strong emphasis on preserving the chemical integrity of key phenolic compounds. To achieve this, key extraction variables, including solvent composition, ultrasound amplitude, and applied energy, were systematically optimized using Response Surface Methodology (RSM). The resulting extracts were characterized by HPLC-ESI-QTOF-MS to maximize both the total phenolic content and extraction yield.

## 2. Materials and Methods

### 2.1. Samples

The *Olea europaea* by-products analyzed (olive leaf and olive pomace) were of the “Hojiblanca” variety, collected in February 2024. These samples were provided by Oleoestepa (Sevilla, Spain). Olive leaves were collected from tree pruning and they were immediately spread out in thin layers on a clean surface in a well-ventilated, shaded area, away from direct sunlight. They were turned regularly to ensure even drying and prevent mold, and the process continued for 20 days until the leaves were completely dried. Olive pomace was generated during the olive oil extraction process in an industrial plant equipped with a hammer crusher, a horizontal blender, one horizontal and one vertical centrifuge (two-phase system), and a conical decanter. This by-product was firstly freezed at −20 °C immediately after olive oil extraction, and then lyophilized using Cryodos equipment (Azbil Telstar SLU, Terrassa, Barcelona, Spain) at a temperature of −50 °C and a pressure of 0.07 mbar. Once both samples were dried, they were ground using a cutting grinder until a homogeneous and fine powder was obtained, and they were stored at −32 °C in darkness until further extraction and analysis for a maximum period of 6 months.

### 2.2. Chemicals

Ethanol was acquired from Scharlab (Barcelona, Spain). Double-deionized water was obtained using a Milli-Q system (Millipore, Bedforf, MA, USA). Standards of *p*-coumaric acid, hydroxytyrosol, and oleuropein were purchased from Sigma-Aldrich (St. Louis, MO, USA). Apigenin was purchased from LGC (Teddington, Middlesex, UK), and luteolin-7-*O*-glucoside, loganin, and verbascoside were purchased from Extrasynthese (Lyon, France).

### 2.3. Ultrasound-Assisted Extraction (UAE) of Polyphenols from Olive Leaves and Olive Pomace

UAE was carried out in an ultrasound extraction system UP400St (Hielscher Ultrasonic GmbH, Teltow, Germany) with a nominal output of 400 W, equipped with an ultrasound probe of 7 mm and performing at 24 kHz. Extracts were prepared by adding 2 g of dried and powdered sample into a vessel with 20 mL of the selected solvent mixture and immersing them in an ice-bath. The extractions were performed in accordance with the experimental design described below. Temperature was monitored throughout the extraction procedure with a final value below 40 °C.

Response Surface Methodology (RSM) was applied in order to optimize the recovery of phytochemical compounds from olive by-products. For this purpose, a Box–Behnken design (BBD) with three central points was used to evaluate the effect of the extraction parameters. The three factors established as independent variables were the percentage of ethanol in the extraction solvent (in a range of 0–100%), ultrasound amplitude (in a range of 30–60 μm), and energy applied to the sample by US (25–100 J∙mL^−1^). The amplitude range between 30 and 60 μm was set on the ultrasonic processor as a percentage, considering that, according to the manual [22], the 7 mm sonotrode used provides an amplitude of 164 μm with a setting of 100%. Therefore, the studied range corresponded to 18–37% amplitude. However, in this study, the amplitude will be expressed in μm, as this parameter is independent of both the sonotrode used and the nominal power of the instrument, unlike amplitude expressed as a percentage. For each variable, three levels were considered, as follows: maximum, medium, and minimum. The response variables were the extraction yield and the phenolic composition of the extracts determined by HPLC analysis, and the objective of the design was to maximize both.

The generated experimental design consisted of a total of 15 experiments that were performed in a randomized order. After ultrasound treatment, samples were cooled at 4 °C and centrifuged at 4430× *g* for 10 min in a centrifuge (FC5718R 230V Serie Frontier™ 5000 Multi Pro, OHAUS Corporation, Parsippany-Troy Hills, NJ, USA), and the supernatant was collected and evaporated at 40 °C to dryness in a rotary evaporator (IKA RV3 eco, IKA-Werke GmbH & Co. KG, Staufen, Germany). After evaporation, the mass of the extract obtained was calculated and reconstituted with the same solvent used for the extraction, up to a concentration of 10 mg∙mL^−1^. Finally, the extracts were filtered (0.45 μm filter) and stored at −32 °C until HPLC analysis. The percentage yield was calculated by the following equation (Equation (1)):

(1) $$\mathrm{Extraction}\mathrm{yield}=\frac{\mathrm{weigh}\mathrm{of}\mathrm{dry}\mathrm{extract}}{\mathrm{weigh}\mathrm{of}\mathrm{dry}\mathrm{sample}}\cdot 100$$

### 2.4. Characterization of Extracts by High-Performance Liquid Chromatography Coupled to Electrospray Ionization Quadrupole Time-of-Flight Mass Spectrometry (HPLC-ESI-QTOF-MS)

The analyses of the obtained extracts were carried out using a previously validated method [23] in a HPLC system coupled with a quadrupole time-of-flight mass spectrometer QTOF-MS (Bruker Daltonik, Bremen, Germany). The QTOF mass analyzer was equipped with an ESI interface operating in negative ion mode. The analytical column used for separation was a Zorbax Eclipse Plus C18, 150 mm × 4.6 mm internal diameter, 1.8 µm (Agilent Technologies, Palo Alto, CA, USA). The mobile phase was water with 0.25% acetic acid (solvent A) and methanol (solvent B) eluted according to the following multi-step gradient: 0 min, 5% solvent B; 7 min, 35% solvent B; 13 min, 45% solvent B; 18.5 min, 50% solvent B; 22 min, 60% solvent B; 29 min, 95% solvent B; 36 min, 5% solvent B. The flow rate was 0.5 mL∙min^−1^ throughout the entire analysis run, the column temperature was maintained at 25 °C, and the injection volume was 10 µL. The MS detection was performed by considering a mass range of 50–1000 *m*/*z*. The optimum values for the source parameters were capillary voltage of +4.5 kV; drying gas temperature, 190 °C; drying gas flow, 9 L∙min^−1^; and nebulizer gas pressure, 2.0 bar. The optimum values for the transfer parameters were capillary output voltage, −150 V; skimmer 1 voltage, −50 V; hexapole 1 voltage, −23 V; hexapole RF, 100 Vpp; and skimmer 2 voltage, −22.5 V. Automatic MS/MS experiments were performed by adjusting the collision energy value to 20 eV. External calibration of the mass spectrometer was performed using sodium acetate solution (5 mM sodium hydroxide in water/2-propanol 1:1 (*v*/*v*), with 0.2% acetic acid) as the calibrant in high precision calibration (HPC) regression mode. The calibration solution was injected at the beginning of each run, and all spectra were calibrated prior to compound identification.

Lastly, the quantitative analysis of the optimized extracts was carried out on the same analytical platform according to the chromatographic and mass spectrometric conditions previously described. Triplicate injections of each extraction replicate for each type of sample were performed to ensure the reproducibility of both the extraction process and the analysis. To carry out the quantitation, calibration curves were prepared with commercially available standards; some of them are present in the extracts and others have similar structures to other identified compounds in the samples. The stock solutions of each of these standards (apigenin, luteolin-7-*O*-glucoside, loganin, verbascoside, oleuropein, hydroxytyrosol, and coumaric acid) were prepared in methanol at a concentration of 1000 mg∙L^−1^ and stored at −20 °C. Then, solutions of these standards were prepared in the same solvent at concentrations of 1, 5, 10, 15, 25, 50, 70, and 100 mg∙L^−1^. The concentration of the phenolic compounds present in the samples was calculated using the individual area of each compound of the chromatogram and by interpolating in the corresponding calibration curve equation of the commercial standard when available or of a structurally similar one. Finally, the limits of detection (LOD) and quantification (LOQ) were determined for each standard compound. The LOD was defined as three times the signal-to-noise ratio (S/N) obtained from a 1 ppm standard solution, whereas the LOQ was defined as ten times the S/N ratio under the same conditions.

Chromatographic and mass spectra data were processed using Data Analysis 6.0 and TASQ software version 2023 (Bruker Daltonik, Bremen, Germany).

### 2.5. Antioxidant Activity: DPPH Assay

The DPPH assay followed a published procedure [24]. The antioxidant activity of extracts was assessed by measuring their ability to scavenge the DPPH radical. The ethanolic solutions of extracts (20 μL) were mixed with 1980 μL of DPPH methanolic solution (40 µg/mL), and incubated for 30 min at room temperature, in the dark. Then, the absorbance was measured at 517 nm against blank (Fisher Scientific Biochrom EZ Read 400, Waltham, MA, USA).

The antioxidant activity (AA) was calculated according to the following formula: (Equation (2)),

(2) $$AA\%=\frac{{ABS}_{DPPH}-{ABS}_{sample}}{{ABS}_{DPPH}}\cdot 100$$

In this scenario, the absorbance of DPPH radical (ABS_DPPH_) and the absorbance of the extract (ABS_sample_) were required to obtain the antioxidant activity (AA).

A calibration curve using Trolox (6-hydroxy-2,5,7,8-tetramethylchroman-2-carboxylic acid) at different concentrations (0–10 mg∙L^−1^) was built and used as a reference and expressed as mg of Trolox equivalent/g of dry extract. All the experiments were performed in triplicate.

### 2.6. Statistical Analysis

The RSM was developed and analyzed using Statgraphics software, version 5.1 (Statgraphics Industries Inc., The Plains, VA, USA). This software was also used to create the model equation and to generate a 3D prediction profiler plot, which helped identify the optimal values of the response variables to maximize both the total phenolic content (TPC) and the extraction yield. The adequacy of the models was checked by the quadratic coefficient of determination (R^2^) and the lack of fit value. Values were considered significantly different when *p* < 0.05.

## 3. Results and Discussion

### 3.1. Characterization of UAE Olive By-Products by HPLC-ESI-QTOF-MS

**Figure 1:**
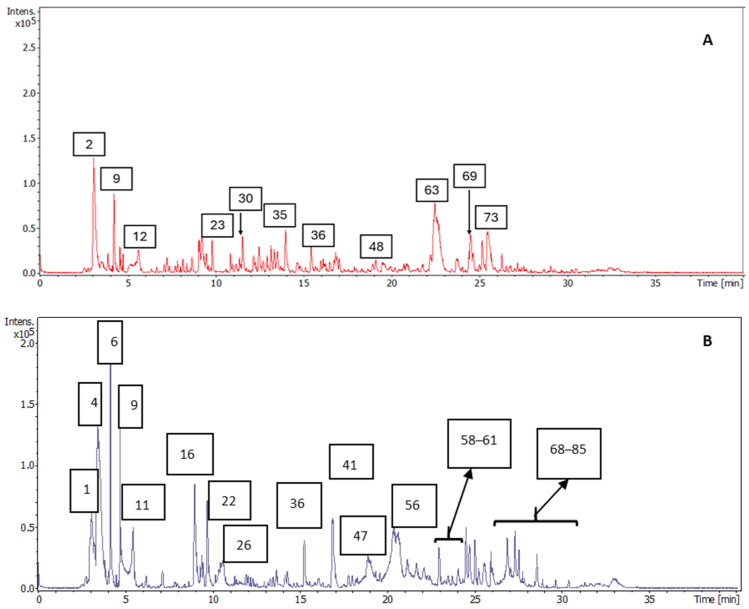
Representative BPC of olive pomace (**A**) and olive leaves (**B**) extracts where the main peaks have been numbered according to their elution order. These numbers correspond to those of Table 1.

Representative base peak chromatograms (BPCs) of olive pomace and olive leaf extracts obtained by UAE are shown in Figure 1. The detected compounds were characterized thanks to the chemical information provided by the HPLC-ESI-QTOF-MS instrument. In this sense, compounds were tentatively identified by interpretation of their mass spectra and the molecular formula provided by the DataAnalysis 6.0 software, together with the information previously reported in the databases and literature concerning olive pomace and leaf composition. Whenever possible, the putative identification was confirmed by a comparison of retention times and MS/MS spectra to commercial standards, while the remaining compounds remain tentatively identified, including several isomers that exhibited the same molecular formula and similar fragmentation patterns but different retention times.

A total of 85 compounds were detected in the analyzed olive pomace and olive leaf extracts, of which 63 of them could be tentatively identified, while the remaining 22 compounds were unknown, despite the efforts made for characterization. All detected compounds are shown in Table 1, which summarizes the following information: peak number assigned, retention time (RT), experimental and theoretical *m*/*z*, error (ppm), molecular formula, milisigma value (mSigma), fragmentation pattern, and the proposed compound for each chromatographic peak. The mSigma value points out the statistical similarity between the theoretical and measured isotopic patterns, in such a way that a low mSigma value means that the experimental isotopic pattern of a peak is very similar to the theoretical isotopic pattern for the proposed molecular formula.

Most of the identified compounds are characteristic of olive-related products and by-products, or are derivative compounds, and as such, they have been extensively described in the vast literature concerning these matrices. As shown in Table 1, a considerable number of compounds belong to or are derived from the secoiridoid family, with oleuropein, ligstroside, and their derivatives being the most widely represented. Relatively unaltered secoiridoids, such as oleuropein and ligstroside, along with their isomers (peaks 43, 62, 68, 74, and 45, 72, respectively), have been found in extracts from both studied matrices. In contrast, more complex derivatives, such as hydroxyoleuropein and its isomers (peaks 37, 38, 40), or dihydrooleuropein (peak 48), were identified only in olive leaves, which is consistent with the previously reported results [25].

On the other hand, a high number of oleuropein aglycone isomers (peaks 57, 58, 60, 67, 69, 70, 73, 75, 78, 80, 83, 84) were exclusively found in olive pomace extracts, as the release of endogenous enzymes during the mechanical crushing of the fruit causes the hydrolysis of oleuropein [26]. A similar number of isomers have been previously described in olive oil, with these species being attributed to the biotransformation of oleuropein aglycone due to the keto-enol tautomeric equilibrium on the exposed hemiacetal carbon after the removal of the glucose moiety, which involves ring opening [27]. The extensive degradation of oleuropein by enzymatic action could explain the presence of elenolic acid and isomers (peaks 29, 34, 47, 76), and some derivatives (peaks 26, 27) exclusively in OP extracts.

Despite the absence of oleuropein aglycone in olive leaf extracts, oleacein (a decarboxymethylated derivative of oleuropein aglycone) was detected in this matrix, although its peak intensity was considerably lower compared to olive pomace extracts. Other related secoiridoids were also identified in both matrices, including oleoside isomers (peaks 20, 33) and its methyl ester derivative (peak 39), as well as glucosylated derivatives of elenolic acid (peaks 16, 22). Iridoids characteristic of olives, such as loganic acid (peak 17), 7-epiloganin (peak 28), and lamiol (peak 36), were also found in both by-products.

Belonging to the group of phenolic alcohols, hydroxytyrosol (peak 23) was detected in both matrices, as expected, along with its glucosylated derivatives (peaks 15 and 22), although only one isomer was found in OL extracts. The presence of these hydroxytyrosol glucoside isomers has been previously reported in olive pomace [28]. In addition, an oxidized form of hydroxytyrosol was identified in OL extracts, which has been previously described in olive by-products, and ethylguaiacol (peak 25) was detected exclusively in olive pomace extracts.

On the other hand, the phenylpropanoid verbascoside (peak 41), as well as its isomer isoverbascoside (peak 53), were found in both matrices, both of which have also been previously described as olive phenolic compounds in other research studies [29]. Several flavonoids were also present in both matrices, mainly luteolin (peak 83) and its different glycosylated derivatives (peaks 46, 50, 52, 55, 65). In contrast, apigenin (peak 85) was detected exclusively in olive pomace extracts, as described by Cecchi et al.

Apart from these phenolic families, a wide variety of organic acids have been identified, mainly in olive pomace extracts, including glucuronic acid (peak 1), citric acid (peak 9), xylonic acid (peak 2), treonic acid (peak 4), quinic acid (peak 5), and malic acid (peak 7), which is consistent with the previous scientific literature related to OP extracts [30].

### 3.2. Optimization of Ultrasound-Assisted Extraction (UAE)

The UAE methodology was optimized following the procedure described in Section 2.3, by applying a Box–Behnken design (BBD) to assess the following three independent variables: ethanol concentration in the extraction solvent, ultrasound amplitude, and energy applied to the sample by US, each at three levels, with three central points. This design resulted in the 15 randomized experiments shown in Table 2. All experiments were conducted with a fixed solid–liquid ratio of 1:10, based on previously optimized conditions reported in other studies, both for olive pomace [31] and for olive leaves [32], where it was demonstrated that this solid-to-solvent ratio was appropriate and minimized both the amount of solvent used and the energy cost and duration of the subsequent solvent removal step. The type of solvent used for extraction was one of the most thoroughly investigated factors. Ethanol and water are the most suitable solvents for the food and nutraceutical industries because they are green and GRAS solvents that enhance the efficiency of phenolic compounds extraction [33]. Furthermore, mixtures of water and ethanol prevent generation of radicals during cavitation, which may lead to the oxidation of bioactive compounds [18]. Therefore, the extraction solvent composition was assessed across a range of ethanol concentrations, from 0 to 100%.

Regarding specific UAE parameters, there is no consensus on which variables are the most influential in the extraction process, and different studies describe the extraction method using different parameters such as power percentage or amplitude percentage, which are not reproducible across different ultrasonic probes. To overcome this limitation, in this study the US power was assessed as an ultrasound amplitude expressed in µm instead of a percentage to facilitate the reproducibility of the optimized conditions across different sonotrodes, making this parameter independent of the ultrasound device used [34]. The range tested was from 30 to 60 μm to ensure the industrial scalability of the optimal conditions since industrial probes can operate at up to 63 μm.

Generally, the scientific literature agrees on the paramount importance of ultrasound intensity in UAE, since extraction yield usually increases with this parameter up to a certain threshold. Most reported studies evaluate US intensity as power density (W∙mL^−1^) due to its independence from vessel geometry and extraction solvent volume, and extraction time as independent factors; however, these parameters are inherently interrelated. An increase in either variable translates into a greater energy delivery to the sample, enhancing cell disruption and promoting compound transfer from the matrix to the solvent. Nevertheless, excessive power density or prolonged extraction time intensifies cavitation, leading to elevated temperatures and potential compound degradation. The relationship between these two variables can be expressed as follows:

Energy input (J∙mL^−1^) = Power density (W∙mL^−1^) ∙ Extraction time (s)

Therefore, in the present study, the third variable chosen for optimization was the specific energy input to the sample by US, measured in J∙mL^−1^, as a single integrated parameter allowing simultaneous optimization of both power density and extraction time. This approach reduces experimental complexity and mitigates the risk of overfitting that may arise from attempting to independently optimize variables exerting overlapping effects on extraction efficiency. The studied range for this variable was 25 to 100 J∙mL^−1^. In this regard, values exceeding 100 J∙mL^−1^ could affect the phenolic composition, leading to some degradation of the bioactive compounds, whereas energies below 25 J∙mL^−1^ are insufficient to rupture the vegetable cell wall, making the extraction incomplete [35].

Table 2 summarizes the values of the independent variables, as well as the resulting total run time and temperature increase for the 15 experiments conducted according to the BBD. The temperature was monitored throughout the extraction procedure. The starting temperature was set at 15 °C. The different combinations of the extraction conditions showed the same variations in the temperature increase for both matrices, with a final value below 40 °C, ensuring that phenolic compounds were not exposed to high temperatures that could cause thermal degradation. Regarding extraction time, all experiments lasted between 1 and 4 min. In this regard, as many authors have noted, this technique does not require long extraction times to obtain elevated contents of phenolic compounds, highlighting the remarkable efficiency of this extraction technique [36,37]. Compared to using an ultrasonic bath for the extraction of phenolic compounds from olive by-products [31], this method requires shorter extraction times and results in lower temperature increases, reducing the risk of degrading certain phenolic compounds and potentially offering additional advantages.

**Figure 2:**
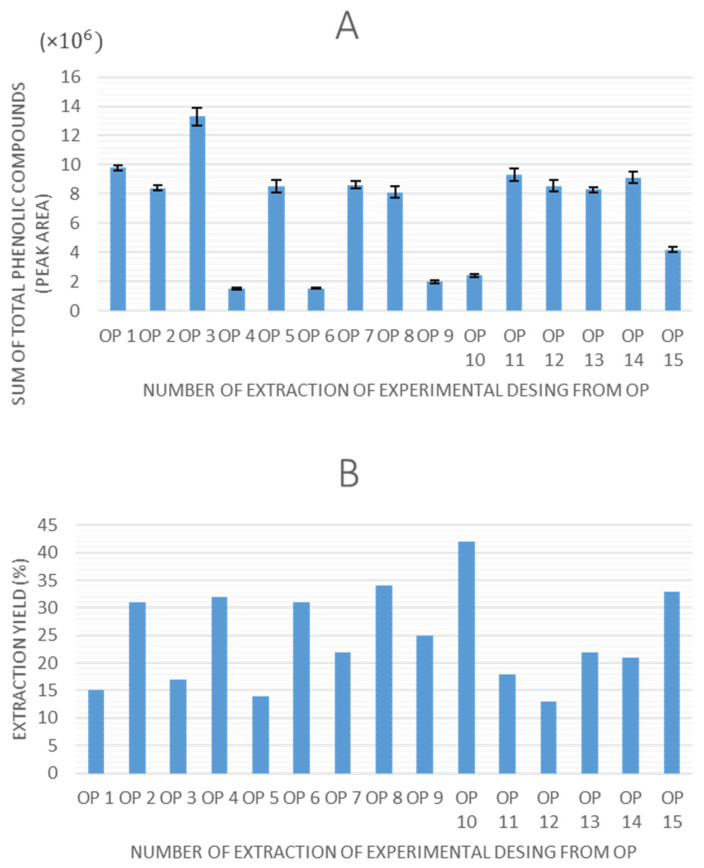
TPC obtained as sum of total peak area of identified phenolic compounds (**A**) and extraction yield (**B**) of the olive pomace extracts (OP).

**Figure 3:**
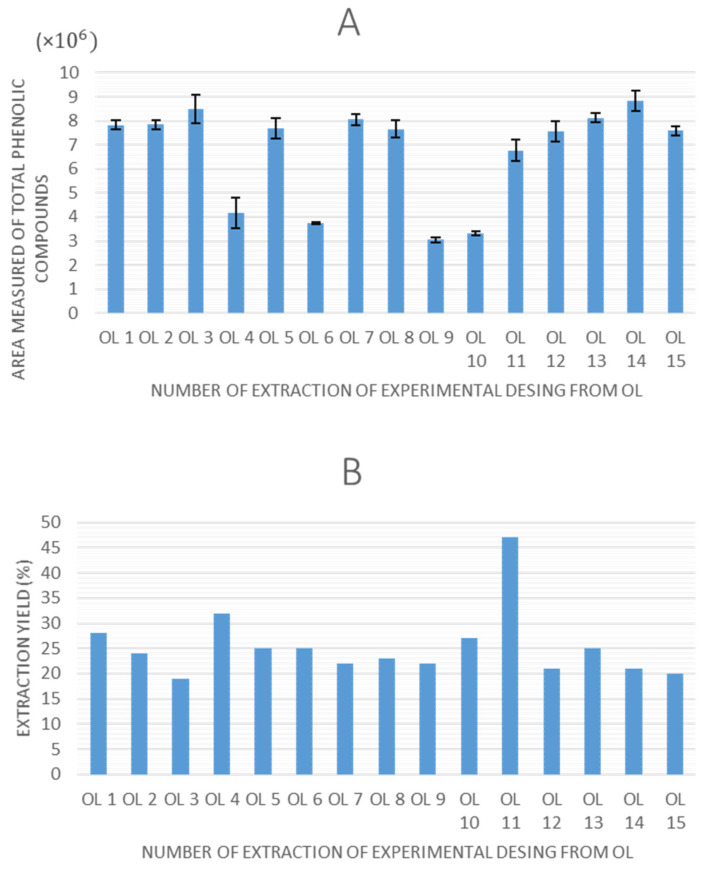
TPC obtained as sum of total peak area of identified phenolic compounds (**A**) and extraction yield (**B**) of the olive leaves extracts (OL).

Once all of the experiments were completed, the extraction yield and total peak area of phenolic compounds obtained by HPLC-ESI-QTOF-MS were selected as response variables to evaluate the experimental designs. The values obtained for these response variables are shown in Figure 2 and Figure 3 for olive pomace and olive leaves, respectively.

Regarding the graphs illustrating the TPC for each experiment using olive pomace and olive leaves, a consistent trend is observed in both matrices, as follows: extractions performed without ethanol in the solvent system (0% ethanol) resulted in lower quantified areas of total phenolic compounds. This indicates that ethanol is a critical factor influencing extraction efficiency in UAE. Moreover, experimental points 7 (8.6 × 10^6^ in OP, 8.2 × 10^6^ in OL), 14 (9.1 × 10^6^ in OP, 8.6 × 10^6^ in OL), and 15 (8.8 × 10^6^ in OP, 7.8 × 10^6^ in OL), which correspond to the central points of the design, demonstrated high repeatability, confirming the precision and stability of the experimental setup.

In terms of extraction yield, in all experiments it ranges between 15–50%, which could be considered a relatively high value, and it is in agreement with the previously reported data for UAE in olive pomace [31,38]. However, no discernible trend is observed that accounts for the variation in yield values among the different experiments across any of the matrices.

The effect of the independent variables (US amplitude, ethanol content of extraction solvent, and specific energy input to the sample by US) on the different response variables (extraction yield and TPC) was evaluated. The experimental results were fitted to second-order polynomial models. To assess the adequacy of the proposed models and the fit of the obtained data, an analysis of variance (ANOVA) was performed for each experimental model corresponding to the different response variables. The analysis included an evaluation of model adequacy, the coefficient of determination (R^2^), and the lack-of-fit test. Table 3 presents the ANOVA results for each model for olive pomace and olive leaves.

Regarding olive pomace, the model built for extraction yield did not present any significant variable, showing that the independent variables within the studied ranges did not affect the extraction yield. Therefore, this model was discarded. On the other hand, ethanol content and its quadratic effect, as well as the quadratic effect of amplitude and the interaction between ethanol content and applied energy, had a significant effect on the total phenolic content. Additionally, the lack-of-fit test and R^2^ values confirmed the suitability of the model.

With respect to olive leaves, as with the case of olive pomace, only the model built for total phenolic content showed a significant influence of the studied independent variables, along with suitable fit and adequacy. In this case, the only variable that demonstrated a significant effect on TPC was the extraction solvent composition, as well as its quadratic effect.

As can be observed, among the assessed variables, the concentration of ethanol in extraction solvent was the most significant factor with the highest impact in both models. The concentration of ethanol is a key factor in ultrasound-assisted extraction (UAE) of phenolic compounds, as it significantly affects solvent polarity, solute–solvent interactions, and the permeability of plant cell membranes. The effectiveness of the extraction process relies heavily on aligning the solvent’s polarity with the wide polarity spectrum of phenolic compounds naturally found in plant materials. Using ethanol–water mixtures enables precise adjustment of polarity, improving the extraction of both water-soluble and moderately non-polar phenolics [39]. In addition, ethanol can disrupt plant tissue structure by increasing membrane permeability and causing protein denaturation, which in turn promotes mass transfer and the release of intracellular compounds. These effects are enhanced by acoustic cavitation generated during UAE, which contributes to physical cell disruption and deeper solvent diffusion [40]. The ethanol-to-water ratio also directly impacts cavitation by altering the solvent’s physical properties, such as vapor pressure, viscosity, and surface tension. Although the ideal ethanol concentration depends on the specific matrix and target compounds, it consistently proves to be the most critical parameter for improving selectivity and maximizing extraction efficiency in UAE of phenolic-rich plant materials [41]. This effect has been previously described in the recovery of phenolic compounds from olive wastes by UAE [42,43], as well as by other extraction techniques [44].

The interaction between ethanol concentration and the energy applied has been shown to have a negative effect on the extraction of total polyphenols in the case of olive pomace. This effect is linked to the enhancement of the cavitation phenomenon produced by a higher amount of applied energy. In this context, this phenomenon causes an increase in free radicals, which accelerates the degradation of the phenolic compounds extracted. However, this effect is partially counteracted by the ability of ethanol to scavenge the generated radicals; hence, their interaction significantly influences TPC levels [45].

Regarding US amplitude, its quadratic effect has a lesser influence on the extraction of total phenolics from olive pomace than the other variables. In this sense, this slight influence on the extraction could be attributed to the previous breakup of the cellular matrix in this sample, as it had already been subjected to an extraction process during beating and crushing to obtain the olive oil [46].

**Figure 4:**
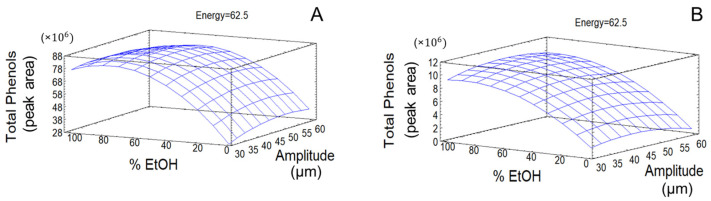
Response surface plots of the models built for TPC in olive leaves (**A**) and olive pomace (**B**).

The response surface plots corresponding to the models built for TPC showed significant effects of studied variables, as well as a suitable fit, and the predictive capacities are shown in Figure 4 for olive pomace and olive leaves.

The fitted simplified equations that explain the behavior of these response variables for olive pomace are displayed below, where A represents US amplitude, B corresponds to ethanol content in extraction solvent, and C denotes applied energy, as follows:

TPC = 3,550,730 + 8555.61 × A + 157,667 × B − 82,109.3 × C − 340.812 × A^2^ − 675.34 × B^2^ − 508.394 × B × C.

In the case of olive leaves, the simplified equations were as follows:

TPC = −786,974.0 + 215,806.0 × A + 156,728.0 × B – 25,284.4 × C − 948.306 × B^2^

In this regard, Table 4 presents the optimal experimental conditions for each model of both by-products, including the predicted values for the response variables.

As can be seen, the optimal parameters to maximize TPC in olive by-products were quite similar, unless EtOH content was present.

Once the optimal conditions for UAE were defined as described above, they were used to obtain both optimal extracts in triplicate, which were then analyzed by HPLC-ESI-QTOF-MS. To validate the designs, the experimentally obtained sum of the total phenolic peak areas were compared to the predicted values, and the results of this validation are presented in Table 5, including the relative error between both values. The results show that the experimental peak areas were very close to the predicted ones, with relative errors below 25%, which may be considered a validation of these models.

It is worth noting that the extraction yields obtained from both by-products are comparable to those previously reported in the literature, despite the short extraction time and mild conditions applied [37,47,48,49]. This is of particular interest, since optimizing TPC in both by-products enables acceptable yields in the development of phenolic-enriched extracts.

The optimized conditions by the experimental design ensure that the resulting extracts are not only produced efficiently, but also genuinely enriched with target compounds. Quantitation of phenolic compounds by HPLC-QTOF-MS determines the meaningful relationships between the total extract yield (which includes the target compounds alongside other components present in the extract) and the relative concentrations of phenolic compounds.

These compounds were quantified using the standard calibration curves described in Section 2. Table S1 (Table attached in Supplementary Materials) presents the calibration equations used to quantify phenolic compounds from olive pomace and olive leaf extracts and the LOD and LOQ determined for each compound.

The chromatographic area of the detected peak for each compound was interpolated into the corresponding calibration curve of the selected standard based on structural similarity, obtaining the calculated concentration. In this way, hydroxytyrosol derivatives were quantified using the hydroxytyrosol standard curve; the luteolin-7-*O*-glucoside curve was used to quantify glycosylated flavonoids; and secoiridoids and its derivatives were quantified using the oleuropein standard curve, while loganin was used as a surrogate standard for iridoids. In these cases, results are expressed as hydroxytyrosol equivalents (HyE), luteolin-7-*O*-glucoside equivalents (LuGE), oleuropein equivalents (OE), or loganin equivalents (LgE), respectively. It should be noted that this is a commonly used approximation, and that the concentration results obtained using calibration curves of structurally similar compounds should only be used for comparative purposes, as the actual concentrations of these compounds may differ. The compound content, expressed in mg of compound/g of dry extract, was calculated for each extract in triplicate, and the resulting data, expressed as the mean value ± standard deviation, are shown in Table 6 and Table 7.

The TPC in the olive pomace extract, calculated as the sum of all of the individually quantified phenolic compounds, was approximately 43 mg TPC/g of dried extract, while the olive leaf extract presented a TPC of around 30 mg/g. These values are consistent with previous studies; for instance, Cioffi [47] reported a similar TPC of approximately 30 mg/g from olive pomace extracts obtained by UAE. Other authors have documented lower concentrations, around 20 mg TPC/g dried extract, in both olive pomace and olive leaf UAE extracts [48,49,50]. In the case of Chanioti and Tzia [50] and Ghomari [48], extractions were carried out over extended periods (4 h) using ultrasonic baths. Conversely, Mojerlou and Elhamirad [49] employed an ultrasonic probe with an extraction time of 3 min, similarly to our optimized conditions, but at a higher temperature (60 °C), which would imply an increase in the energetic cost of the process.

Evaluation of the individual compound concentrations revealed that oleuropein and its isomers were present at approximately 10 mg/g in both samples, a level higher than previously reported in other studies. Those studies used solid–liquid extraction and PLE methods, which involve more severe conditions that may lead to the degradation of phenolic compounds [23,26,28,29].

Degradation products derived from oleuropein were also evaluated, including oleuropein aglycone. In the case of olive pomace, this compound is formed as a result of the mechanical processes involved in olive oil production. During this process, glycosylated forms are hydrolyzed by endogenous enzymes, leading to the formation of aglycone derivatives. A total of 12 isomers of oleuropein aglycone were identified in the optimal olive pomace extract, reaching concentrations exceeding 15 mg/g of dried extract, higher than those previously reported [47,51]. In contrast, oleuropein aglycone was not detected in olive leaf extract, likely due to the absence of mechanical processing, which limits the enzymatic hydrolysis of glycosidic bonds [29]. Since this derivative could also be formed during the extraction process, when aggressive conditions such as high-temperature or long extraction time are applied, this absence suggests a lower level of degradation in the olive leaves matrix compared to what has been described in previous studies [51,52,53].

Oleacein, another compound formed from oleuropein degradation, was also one of the main compounds quantified in olive pomace extract. Rosa reported an oleacein concentration of 7.85 mg/g in an extract obtained by UAE [53], while in the present study, the olive pomace extract showed a concentration of 3.1 ± 0.1 mg/g. This occurred despite the higher oleuropein content in this matrix.

Hydroxytyrosol was also quantified in both extracts. In olive pomace, its concentration was 0.50 ± 0.05 mg/g of dried extract, whereas in olive leaves, it was 0.148 ± 0.004 mg/g. These values differ from those reported in other studies. For instance, conventional extraction and PLE of olive pomace have yielded extracts with hydroxytyrosol contents approximately 3 and nearly 20 times higher, respectively [29]. Similarly, the UAE of olive leaves has been reported to produce hydroxytyrosol levels of around 15 mg/g, with oleuropein not being detected [48]. The relatively low concentration observed in this study suggests a limited hydrolysis of oleuropein.

Oxidized hydroxytyrosol was detected exclusively in the olive leaf extract, only at trace levels, below the LOQ. This oxidized derivative typically arises from the hydrolysis and subsequent oxidation of oleuropein or oleuropein aglycone under harsh conditions, such as elevated temperatures or prolonged exposure [23]. It is also frequently found in olive pomace, olive paste, and even in olive oil as a result of degradation occurring during olive oil production [1]. However, its presence solely in olive leaf extracts, and not in any of the 15 olive pomace extracts prepared during optimization, despite identical extraction conditions, suggests that this derivative was formed through hydroxytyrosol oxidation prior to extraction. This is likely attributable to the extended ambient-temperature drying process used for olive leaves, as opposed to the rapid, low-temperature freeze-drying employed for olive pomace, which helps preserve phenolic compounds.

The relatively low hydroxytyrosol concentration, combined with only trace levels of its oxidized form in the olive leaf extract, together with the higher abundance of oleuropein and its derivatives, indicates that the optimized UAE conditions—characterized by low temperatures and short extraction times—effectively preserve the phenolic profile of the original matrix.

According to Xie et al., ultrasound intensity—controlled in this study as US amplitude and energy applied to the sample—plays a crucial role in the extraction of phenolic compounds [54]. Low intensities may lead to insufficient cell disruption, resulting in reduced extraction efficiency, whereas higher intensities can enhance recovery, but may also induce compound degradation due to increased temperature. Therefore, this study highlights the importance of carefully balancing ultrasound parameters to maximize phenolic yield while minimizing degradation. The conditions optimized in the present study, significantly milder than those reported in prior studies [37,38,42], appear to represent an effective compromise, achieving substantial extraction with minimal deterioration of bioactive compounds.

Finally, antioxidant activity of optimal olive pomace and olive leaf extracts was assessed by a DPPH assay in triplicate. The results, expressed in mg Trolox equivalents per gram of dry extract, are shown in Table 8.

As can be observed, both optimal extracts exhibit comparable levels of antioxidant activity, with a slightly higher value for olive leaf extract. This contrasts with the previously discussed total phenolic content, which was somewhat higher in the olive pomace extract. This discrepancy may be explained by the greater antioxidant capacity of specific compounds, such as oleuropein and flavonoids, including luteolin and its derivatives, which were found in higher concentrations in olive leaves than in pomace and significantly contribute to the antioxidant activity of olive by-products [55]. The potential synergistic effect of other types of compounds presents in olive leaves, which may have been co-extracted along with phenolic compounds, but whose presence has not been studied, should also not be ruled out. This could be the case for the pentacyclic triterpenic acids oleanolic and maslinic, whose antioxidant capacity against the DPPH radical has been previously demonstrated [56].

Comparing these values to those reported in previous studies is challenging due to the wide variety of in vitro antioxidant activity protocols and the lack of standardized units for reporting results. However, some studies have expressed their findings in the same units employed in this work, facilitating direct comparison. For instance, conventional extraction of olive leaves has yielded extracts with substantially lower antioxidant activity, around 2 mg Trolox equivalents/g dry extract [57]. In contrast, the use of more efficient techniques, such as microwave-assisted extraction, has resulted in extracts with antioxidant capacities of up to 90 mg Trolox equivalents/g dry extract [58].

Regarding olive pomace, UAE using a 50:50 acetone–water mixture as its. extraction solvent has produced extracts with notably higher antioxidant activity, achieving DPPH values of approximately 400 mg Trolox equivalents/g dry extract [59]. Nevertheless, in 2024 Vittorio Carlucci et al. reported antioxidant activities of 63.70 ± 6.03 mg Trolox equivalents per gram of dry extract when using an ultrasonic-assisted extraction (UAE) with water, and 65.92 ± 1.62 mg Trolox equivalents per gram of dry extract with a UAE water/ethanol (15%) extraction [60]. In comparison, the antioxidant activity found in this study indicates that using optimal conditions can achieve higher antioxidant activity than what has been previously reported.

## 4. Conclusions

This study highlights the substantial potential of olive oil by-products, such as olive leaves and pomace, as valuable sources of bioactive compounds, including hydroxytyrosol, oleacein, and oleuropein, and demonstrates the effectiveness of UAE as a sustainable and efficient method for recovering these compounds using environmentally friendly solvents. Response surface models indicate that among the variables studied, ethanol concentration emerged as the most critical factor in enhancing extraction efficiency and preserving phenolic compounds, while minimizing degradation caused by cavitation phenomena. The optimized UAE process was shown to reduce phenolic degradation compared to traditional extraction methods, as evidenced by the high oleuropein and a concurrent reduction in degradation-derivative compounds of this secoiridoid in the extracts. Additionally, both optimized extracts exhibited strong antioxidant activity, as measured by the DPPH assay. These findings emphasize the opportunity to revalorize olive oil by-products within a circular economy framework, converting waste into high-value resources for the food, pharmaceutical, and cosmetic industries. Furthermore, UAE proves to be a reliable and sustainable method for compound recovery, offering shorter extraction times and lower thermal degradation.

## Figures and Tables

**Table 1:** Tentatively identified compounds in olive pomace and olive leaf extracts.

Peak	Proposed Compound	Molecular Formula	RT (min)	*m*/*z* Experimental	*m*/*z* Theorical	Error (ppm)	mSigma	MS/MS	Extracts
**1**	Glucuronic acid	C_6_H_10_O_7_	3.1	193.0352	193.0354	0.7	9.7	193.0356 (100), 165.0407 (89.5), 135.0299 (82.2), 181.0717 (78.7)	OP
**2**	Xylonic acid	C_5_H_10_O_6_	3.1	165.0406	165.0406	−1.0	0.8	-	OL
**3**	Unknown 1	C_6_H_14_O_6_	3.1	181.0725	181.0718	−4.0	3.3	135.0301 (100), 181.072 (41.8), 165.0408 (38.9), 179.0562 (23.5)	OP
**4**	Treonic acid	C_4_H_8_O_5_	3.2	135.0366	135.0299	−0.5	3.7	75.0099 (100), 135.0297 (57.98), 59.0155 (14.97), 89.0254 (13.53)	OP
**5**	Quinic acid	C_7_H_12_O_6_	3.5	191.0558	191.0561	1.6	5.5	191.056 (100)	OP
**6**	Unknown 2	C_13_H_26_O_13_	3.9	387.1153	387.1144	−2.2	10.3	683.2246 (100), 341.108 (81.2), 387.1134 (81.2), 149.0453 (56.1)	OP
**7**	Malic acid	C_4_H_6_O_5_	4.2	133.0137	133.0142	4	0.5	133.0141 (100), 115.0037 (28.4)	OP
**8**	Unknown 3	C_12_H_20_O_11_	4.5	377.0762	377.0776	4.4	15.6	-	OP
**9**	Citric acid isomer 1	C_6_H_8_O_7_	4.7	191.0195	191.0197	1	6.1	191.0197 (100), 111.0086 (32.6)	OP
**10**	Unknown 4	C_7_H_8_O_7_	5.2	203.0195	230.0197	1.3	0.9	203.0197 (100)	OP
**11**	Citric acid isomer 2	C_6_H_8_O_7_	5.5	191.0192	191.0197	3	3.5	191.0197 (100),111.0083 (35.2)	OP *, OL
**12**	3,4-Dihydroxybenzeneacetaldehyde (oxidized hydroxytyrosol)	C_8_H_8_O_3_	5.8	152.0403	152.0401	−1.2	7.2	123.045 (100), 108.0219 (19.07)	OL
**13**	Unknown 5	C_21_H_32_O_16_	6.2	539.1624	539.1618	−1.1	10	539.1612 (100)	OP
**14**	Unknown 6	C_16_H_26_O_11_	7.2	393.1399	393.1402	0.9	6.8	393.14 (100)	OP *, OL
**15**	Hydroxytyrosol glucoside isomer 1	C_14_H_20_O_8_	9.0	315.1085	315.1085	−0.5	9.8	315.1079 (100)	OP *, OL
**16**	Glucopyranosyl acyclodihydroelenolic acid isomer 1	C_17_H_28_O_11_	9.08	407.1570	407.1559	−2.7	16.3	815.3176 (100), 407.1548 (83.3), 153.0551 (25.7)	OP *, OL
**17**	Loganic acid	C_16_H_24_O_10_	9.3	375.1283	375.1297	3.7	26.6	375.1282 (55.7), 355.0669 (27)	OP *, OL
**18**	Unknown 7	C_19_H_28_O_12_	9.3	447.1516	447.1508	−1.8	18.6	447.1515 (100), 355.0653 (34.9), 375.13 (24.7)	OP
**19**	Hydroxytyrosol glucoside isomer 2	C_14_H_20_O_8_	9.4	315.1087	315.1085	−0.4	5.1	315.1078 (100)	OP
**20**	Oleoside isomer 1	C_16_H_22_O_11_	9.4	389.1082	389.1089	1.8	8.7	59.0146 (100), 121.0656 (54.19), 183.0674 (47.72), 101.0233 (47.22), 95.0486 (37.08), 89.0253 (31.84), 71.0142 (31.04)	OL
**21**	Unknown 8	C_19_H_28_O_12_	9.5	447.1503	447.1508	1.1	11.7	447.1493 (100), 315.1093 (29.3)	OP
**22**	Glucopyranosyl acyclodihydroelenolic acid isomer 2	C_17_H_28_O_11_	9.7	407.1556	407.1559	0.6	7.3	815.3189 (100), 407.1555 (85), 81	OP *, OL
**23**	Hydroxytyrosol **	C_8_H_10_O_3_	9.7	153.0551	153.0557	4.1	12.5	-	OP *, OL
**24**	Unknown 9	C_22_H_36_O_15_	9.8	539.1965	539.1981	3.1	12.8	407.1553 (100), 539.1965 (37.9), 153.0558 (36.4)	OP
**25**	4-Ethylguaiacol	C_9_H_12_O_2_	10.2	151.0763	151.0765	1.20	99.2	151.0765 (51.7), 69.034 (43.6), 59.0133 (42)	OP
**26**	Dialdehydic form of decarboxymethylated elenolic acid	C_9_H_12_O_4_	10.5	183.0658	183.0663	2.8	8.3	183.0663 (100), 69.0339 (58.4), 59.0129 (56.5)	OP
**27**	Hydroxylated product of the dialdehydic form of decarboxymethylated elenolic acid	C_9_H_12_O_5_	10.8	199.0598	199.0612	6.9	10.6	59.013 (53.7), 69.0342 (50.5), 183.0663 (48.3), 199.0612 (42.3)	OP
**28**	7-Epiloganin	C_17_H_26_O_10_	11.4	389.1445	389.1453	2.2	6.5	389.1443 (100), 241.0717 (40.2), 95.0501 (24.6), 127.0392 (22.2)	OP *, OL
**29**	Elenolic acid isomer 1	C_11_H_14_O_6_	11.5	241.0714	241.0718	1.6	7	241.0713 (100), 127.0398 (45.2), 95.0501 (45.1)	OP
**30**	Unknown 10	C_18_H_28_O_12_	12.3	435.1522	435.1508	−3.1	10.9	389.1455 (100), 435.1495 (88.8), 390.1464 (28.91), 313.1295 (27.05)	OL
**31**	Unknown 11 isomer 1	C_21_H_30_O_13_	12.5	489.1609	489.1614	1.0	16.5	-	OL
**32**	Unknown11 isomer 2	C_21_H_30_O_13_	12.7	489.1621	489.1614	−1.6	15.0	-	OL
**33**	Oleoside isomer 2	C_16_H_22_O_11_	12.9	389.1077	389.1089	3.1	4	389.1082 (100), 241.0697 (23.9)	OP *, OL
**34**	Elenolic acid isomer 2	C_11_H_14_O_6_	13.7	241.0715	241.0718	1.1	9.2	241.0712 (100), 127.0397 (52.3), 95.05 (50.2)	OP
**35**	Hydroxydecarboxymethyl oleuropein aglycone	C_17_H_20_O_7_	14.21	435.1529	435.1508	−4.9	14.2	435.1508 (100), 241.0716 (29.3)	OP * OL
**36**	Lamiol	C_16_H_26_O_10_	15.2	377.1430	377.1453	1.1	3.4	377.1448 (100)	OP *, OL
**37**	Hydroxyoleuropein isomer 1	C_25_H_32_O_14_	16.2	555.1715	555.1719	0.8	16.4	-	OL
**38**	Hydroxyoleuropein isomer 2	C_25_H_32_O_14_	16.3	555.1715	555.1719	0.8	16.4	-	OL
**39**	Oleoside methylester	C_17_H_24_O_11_	16.83	403.1223	403.1246	5.7	23	403.1255 (100)	OP *, OL
**40**	Hydroxyoleuropein isomer 3	C_25_H_32_O_14_	16.9	555.1697	555.1719	4.1	3.0	-	OL
**41**	Verbascoside **	C_29_H_36_O_15_	16.99	623.1972	623.1981	1.5	5	623.1973 (100)	OP *, OL
**42**	*p*-Coumaric acid **	C_9_H_8_O_3_	17.13	163.0402	163.0401	−1	57.4	-	OP
**43**	Oleuropein isomer 1	C_25_H_32_O_13_	17.79	539.2035	539.1770	2	12.5	539.1774 (100)	OP
**44**	Unknown 12	C_31_H_40_O_16_	18.0	667.2230	667.2244	2	31.4	667.2239 (100), 539.179 (45), 668.2273 (29.9), 523.1814 (28.8), 241.0713 (21.1)	OP
**45**	Ligstroside isomer 1	C_25_H_32_O_12_	18.2	523.1826	523.1821	−1	10.7	523.1817 (100), 241.072 (37.8), 254.1868 (25.9)	OP *, OL
**46**	Luteolin rutinoside isomer 1	C_27_H_32_O_12_	18.7	593.1517	593.1512	−0.9	29.4	241.0719 (100), 593.1492 (48.1), 95.0506 (38.1), 127.0406 (36.1), 539.1794 (31.7)	OP *, OL
**47**	Elenolic acid isomer 3	C_11_H_14_O_6_	18.91	241.0712	241.0718	2.3	3.8	241.0713 (100), 95.1496 (29.4), 127.0398 (27.4)	OP
**48**	Dihydrooleuropein	C_25_H_36_O_13_	19.2	543.2088	543.2083	−0.9	5.9	377.147 (100), 513.1965 (99.82), 525.1967 (92.97), 389.1471 (66.57), 543.2065 (57.03), 71.0144 (38.85), 407.1532 (38.71), 101.0242 (38.46), 313.1296 (32.66)	OL
**49**	Luteolin glucoside isomer 1	C_21_H_20_O_11_	19.3	447.0932	447.0933	0.20	20.8	447.0925 (100), 241.0717 (86.6), 95.0498 (36.4), 127.0401 (34.7)	OP *, OL
**50**	Luteolin rutinoside isomer 2	C_27_H_30_O_15_	19.3	593.1505	593.1512	1.2	25.9	593.15 (100), 241.0718 (92.4), 319.1179 (43.8), 95.05 (43.5), 127.0411 (37.5)	OP
**51**	Unknown 13	C_19_H_32_O_9_	19.3	403.1965	403.1974	2.1	25.1	-	OL
**52**	Luteolin−7-*O*-glucoside **	C_21_H_20_O_11_	19.47	447.0939	447.0933	−1.4	6.8	241.0722 (100), 319.1165 (46.6), 95.0497 (44.6), 127.0404 (40.8), 139.0393 (30.3), 447.0939 (29.9), 183.065 (29.7)	OP *, OL
**53**	Isoverbascoside	C_29_H_36_O_15_	19.59	623.1976	623.1981	0.9	17.8	623.198 (100), 447.0934 (39.9), 241.072 (38.4), 624.2032 (24.9), 95.0503 (20.7)	OP *, OL
**54**	Oleacein	C_17_H_20_O_6_	20.53	319.1176	319.1187	3.4	12.5	319.118 (100), 195.0661 (20.2)	OP *, OL
**55**	Luteolin-7-*O*-diglucoside	C_27_H_30_O_16_	20.7	609.1454	609.1461	1.1	30.9	319.1189 (100), 609.1461 (44), 195.0667 (249, 351.1434 (21.4), 320.1224 (20.9)	OP *, OL
**56**	Unknown 14	C_22_H_40_O_13_	20.9	511.2381	511.2396	2.9	32.2	-	OL
**57**	Oleuropein aglycone isomer 1	C_19_H_22_O_8_	21.15	377.1235	377.1242	1.9	26	377.1238 (100), 241.0718 (27.6), 95.0503 (16.4)	OP
**58**	Oleuropein aglycone isomer 2	C_19_H_22_O_8_	21.66	377.1239	377.1242	0.8	18.8	319.1178 (100), 377.1233 (49.7), 195.0661 (22.3)	OP
**59**	Unknown 15	C_27_H_30_O_14_	21.9	577.1539	577.1563	4.2	47.8	269.0449 (100), 417.1556 (57.2), 270.0484 (19.54), 577.1557 (18)	OL
**60**	Oleuropein aglycone isomer 3	C_19_H_22_O_8_	22.09	377.1240	377.1242	0.4	29.2	377.1228 (100), 241.0709 (22.8)	OP
**61**	Oleuropein diglucoside	C_31_H_42_O_18_	22.35	701.2318	701.2298	−2.7	26	319.1179 (100), 639.2426 (64.3), 381.1553 (24.1), 640.2477 (23.9), 195.066 (21)	OP *, OL
**62**	Oleuropein **	C_25_H_32_O_13_	22.43	539.1765	539.1770	1	37.5	539.1762 (100), 377.1219 (52.8)	OP *, OL
**63**	Unknown 16	C_25_H_44_O_13_	22.8	551.2705	551.2709	0.8	32.7	-	OL
**64**	Unknown 17	C_25_H_28_O_14_	23.0	551.1390	551.1406	2.9	10.4	551.1392 (100)	OP
**65**	Luteolin glucoside isomer 2	C_21_H_20_O_11_	23.50	447.0945	447.0933	−2.8	6.6	447.0926 (100), 363.1446 (43.9)	OP *, OL
**66**	Pinoresinol	C_20_H_22_O_6_	23.91	447.1144	447.0933	1.5	20	-	OP
**67**	Oleuropein aglycone isomer 4	C_19_H_22_O_8_	24.06	377.1240	377.1242	0.5	5.3	377.1222 (100)	OP
**68**	Oleuropein isomer 2	C_25_H_32_O_13_	24.5	539.1765	539.1770	1	37.5	539.1761 (100),303.1245 (47.7), 349.1287 (45.3), 377.1235 (32.8)	OP
**69**	Oleuropein aglylcone isomer 5	C_19_H_22_O_8_	24.5	377.1233	377.1242	2.2	15.8	377.1242 (100)	OP
**70**	Oleuropein aglycone isomer 6	C_19_H_22_O_8_	24.71	377.1248	377.1242	−1.5	14.2	377.1246 (100)	OP
**71**	Unknown 18	C_25_H_42_O_13_	25.1	549.2523	549.2553	5.4	13.5	-	OL
**72**	Oleuropein aglycone isomer 7	C_19_H_22_O_8_	25.25	377.1231	377.1242	2.9	19.9	377.1231 (100)	OP
**73**	Oleuropein isomer 3	C_25_H_32_O_13_	25.4	539.1768	539.1770	1.2	19.3	377.1239 (100), 539.1756 (30.3), 241.0727 (25), 378.1288 (24.6)	OP *, OL
**74**	Oleuropein aglycone isomer 8	C_19_H_22_O_8_	25.57	377.1239	377.1242	0.7	17.4	377.1242 (100), 307.0827 (14.9), 275.0913 (14.2)	OP
**75**	Elenolic acid isomer 4	C_11_H_14_O_6_	25.95	241.0709	241.0718	3.6	15.4	241.0714 (100), 95.0503 (27.5),127.0405 (23.9)	OP
**76**	Unknown 19	C_25_H_28_O_13_	26.0	535.1459	535.1457	−0.3	21	535.145 (100), 377.123 (96.7), 378.1281 (23.3), 307.0823 (20.9)	OP
**77**	Oleuropein aglycone isomer 9	C_19_H_22_O_8_	26.02	377.1242	377.1242	0	14.9	377.1233 (100)	OP
**78**	Unknown 21	C_27_H_38_O_15_	26.3	601.2121	601.2138	2.8	31.0	-	OL
**79**	Oleuropein aglycone isomer 10	C_19_H_22_O_8_	26.87	377.1238	377.1242	1	10.6	377.1243 (100), 241.0716 (39.6), 307.0836 (21), 95.051 (17.2)	OP
**80**	Unknown 22	C_26_H_36_O_13_	27.0	555.2359	555.2083	1.4	14.8	555.2086 (100), 377.1246 (59.2)	OP
**81**	Luteolin	C_15_H_10_O_6_	27.42	285.0534	285.0405	1.6	8.4	285.0399 (100)	OP *, OL
**82**	Oleuropein aglycone isomer 11	C_19_H_22_O_8_	27.55	377.1240	377.1242	0.5	2.2	377.1239 (100)	OP
**83**	Oleuropein aglycone isomer 12	C_19_H_22_O_8_	28.57	377.1241	377.1242	0.2	14	377.1238 (100)	OP
**84**	Apigenin **	C_15_H_10_O_5_	28.58	265.1481	265.1445	−13.5	41.6	265.1484 (100), 100.934 (10.8)	OP
**85**	Unknown 22	C_17_H_26_O_4_	30.40	293.1750	293.1758	3	15.6	293.175 (100), 236.1067 (12.7)	OP

RT (retention time); OP: Olive Pomace; OL: Olive Leaves; * Chromatographic and MS data obtained from the marked extract. ** Identification confirmed using commercial standards.

**Table 2:** Box–Behnken design applied to olive pomace and olive leaves.

Experiment	Independent Variables			Operational Parameters Monitoring	
	Amplitude (µm)	%Ethanol	Energy (J∙mL^−1^)	Time (s)	∆T (°C)
**1**	45	100	100	140	19
**2**	30	50	100	160	20
**3**	45	100	25	110	17
**4**	45	0	25	160	20
**5**	30	50	25	150	20
**6**	60	0	62.5	200	22
**7**	45	50	62.5	170	21
**8**	60	50	25	110	17
**9**	30	0	62.5	220	23
**10**	45	0	100	230	23
**11**	60	100	62.5	100	17
**12**	30	100	62.5	120	18
**13**	60	50	100	140	19
**14**	45	50	62.5	160	20
**15**	45	50	62.5	160	20

**Table 3:** Analysis of variance (ANOVA) of olive pomace and olive leaves models for each response variable.

SOURCE	OLIVE POMACE		OLIVE LEAVES	
	Yield	TPC	Yield	TPC
	*p*-Value		*p*-Value	
**A: AMPLITUDE**	0.4167	0.7437	0.0168 ^a^	0.9337
**B: ETHANOL**	0.0612	0.0005 ^a^	0.1368	0.0117 ^a^
**C: ENERGY**	0.3364	0.0744	0.2476	0.6733
**AA**	0.8196	0.0186 ^a^	0.0621	0.2711
**AB**	0.9460	0.1552	0.0151 ^a^	0.3547
**AC**	0.1866	0.6234	0.4440	0.8319
**BB**	0.6175	0.0027 ^a^	0.0167 ^a^	0.0186 ^a^
**BC**	0.8890	0.0136 ^a^	0.0319 ^a^	0.8947
**CC**	1.2321	0.0835	0.4158	0.6577
**LACK-OF-FIT**	0.0018 ^a^	0.0503	0.0208 ^a^	0.6458
**R2**	0.61	0.97	0.61	0.97

R^2^: coefficient of determination; TPC: total phenolic compounds; ^a^ significant (*p* < 0.050).

**Table 4:** Optimal conditions of the models and predicted values of the response variables of olive by-products.

Olive by-Product	Model	Amplitude (µm)	EtOH (%)	Energy (J∙mL^−1^)	Predicted TPC Value (Peak Area)
**Olive Pomace**	TPC	46	100	25	1.23 × 10^7^
**Olive Leaves**	TPC	42	72	25	8.88 × 10^6^

**Table 5:** Validation of models from olive by-products extracts.

Olive by-Product	Model	Yield (%)	Experimental TPC (Peak Area, Mean ± SD)	Predicted TPC (Peak Area)	Relative Error (%)	CV * (%)
**Olive Pomace**	TPC	15 ± 4	1.35 × 10^7^ ± 1.26 × 10^5^	1.23 × 10^7^	9.9	0.93
**Olive Leaves**	TPC	22 ± 2	8.30 × 10^6^ ± 4.48 × 10^4^	8.88 × 10^6^	6.50	0.53

* CV: Coefficient of variance.

**Table 6:** Quantification of olive pomace optimal extract.

RT (min)	Proposed Compound	mg/g Dry Extract
**9.00**	Hydroxytyrosol glucoside isomer 1	0.22 ± 0.02 ^a^
**9.08**	Glucopyranosyl acyclodihydroelenolic acid isomer 1	1.54 ± 0.08 ^b^
**9.08**	Glucopyranosyl acyclodihydroelenolic acid isomer 2	<LOQ
**9.30**	Loganic acid	<LOQ
**9.40**	Hydroxytyrosol glucoside isomer 2	<LOQ
**9.70**	Hydroxytyrosol	0.50 ± 0.05
**10.20**	4-Ethylguaiacol	<LOQ
**10.50**	Dialdehydic form of decarboxymethylated elenolic acid	2.1 ± 0.1 ^b^
**10.80**	Hydroxylated product of the dialdehydic form of decarboxymethylated elenolic acid	<LOQ
**11.40**	7-Epiloganin	<LOQ
**11.50**	Elenolic acid isomer 1	1.3 ± 0.1 ^b^
**12.90**	Oleoside isomer 1	<LOQ
**13.70**	Elenolic acid isomer 2	<LOQ
**14.21**	Hydroxydecarboxymethyl oleuropein aglycone	0.119 ± 0.002 ^b^
**15.20**	Lamiol	0.275 ± 0.008 ^c^
**16.83**	Oleoside methylester	<LOQ
**16.99**	Verbascoside	6.6 ± 0.5
**17.13**	Coumaric acid	0.090 ± 0.005
**17.79**	Oleuropein isomer 1	0.246 ± 0.004 ^b^
**18.20**	Ligstroside isomer 1	0.89 ± 0.06 ^b^
**18.70**	Luteolin rutinoside isomer 1	<LOQ
**18.91**	Elenolic acid isomer 3	<LOQ
**19.30**	Luteolin rutinoside isomer 2	<LOQ
**19.47**	Luteolin-7-*O*-glucoside	0.146 ± 0.001
**19.59**	Isoverbascoside	<LOQ
**20.53**	Oleacein	3.1 ± 0.1 ^b^
**21.15**	Oleuropein aglycone isomer 1	3.10 ± 0.02 ^b^
**21.66**	Oleuropein aglycone isomer 2	3.16 ± 0.01 ^b^
**22.09**	Oleuropein aglycone isomer 3	2.251 ± 0.004 ^b^
**22.35**	Oleuropein diglucoside	1.90 ± 0.03 ^b^
**22.43**	Oleuropein	5.01 ± 0.04
**23.50**	Luteolin glucoside isomer	<LOQ
**24.06**	Oleuropein aglycone isomer 4	1.28 ± 0.04 ^b^
**24.50**	Oleuropein isomer 2	1.55 ± 0.02 ^b^
**24.50**	Oleuropein aglycone isomer 5	1.28 ± 0.04 ^b^
**24.71**	Oleuropein aglycone isomer 6	0.43 ± 0.01 ^b^
**25.17**	Ligstroside isomer 2	<LOQ
**25.25**	Oleuropein aglycone isomer 7	0.18 ± 0.04 ^b^
**25.40**	Oleuropein isomer 3	<LOQ
**25.57**	Oleuropein aglycone isomer 8	0.39 ± 0.07 ^b^
**26.02**	Oleuropein aglycone isomer 9	2.5 ± 0.2 ^b^
**26.87**	Oleuropein aglycone isomer 10	<LOQ
**27.42**	Luteolin	2.26 ± 0.08 ^d^
**27.55**	Oleuropein aglycone isomer 11	<LOQ
**28.57**	Oleuropein aglycone isomer 12	1.10 ± 0.07 ^b^
**28.58**	Apigenin	<LOQ

^a^ mg HyE/g dry extract; ^b^ mg OE/g dry extract; ^c^ mg LgE/g dry extract; ^d^ mg LuGE/g dry extract.

**Table 7:** Quantification of olive leaves optimal extract.

RT (min)	Proposed Compound	mg/g Dry Extract
**5.8**	3,4-Dihydroxybenzeneacetaldehyde (oxidized hydroxytyrosol)	<LOQ
**8.5**	Glucopyranosyl acyclodihydroelenolic acid isomer	<LOQ
**8.6**	Hydroxytyrosol glucoside isomer 1	0.010 ± 0.003 ^a^
**8.7**	Oleoside	0.91 ± 0.03 ^b^
**8.9**	Loganic acid	0.27 ± 0.01 ^c^
**9.1**	Hydroxytyrosol glucoside isomer 2	0.444 ± 0.009 ^a^
**9.2**	4-Ethylguaiacol	<LOQ
**9.3**	Hydroxytyrosol	0.148 ± 0.004
**9.5**	Glucopyranosyl acyclodihydroelenolic acid isomer 2	<LOQ
**11.0**	7-Epiloganin	<LOQ
**12.4**	Oleoside isomer 2	0.36 ± 0.02 ^b^
**14.7**	Lamiol	0.67 ± 0.03 ^c^
**15.2**	Hydroxyoleuropein isomer 1	<LOQ
**16.2**	Hydroxyoleuropein isomer 2	<LOQ
**16.3**	Verbascoside	0.849 ± 0.002
**17.2**	Hydroxyoleuropein isomer 3	<LOQ
**17.9**	Luteolin rutinoside isomer 1	0.420 ± 0.005 ^d^
**18.1**	Dihydroleuropein	0.19 ± 0.01 ^b^
**18.4**	Luteolin-7-*O*-glucoside	1.901 ± 0.001
**18.5**	Isoverbascoside	0.3472 ± 0.0004
**18.6**	Luteolin rutinoside isomer 2	<LOQ
**19.5**	Luteolin-7,4-*O*-diglucoside/rutin	0.1517 ± 0.0005 ^d^
**19.8**	Oleacein	<LOQ
**21.4**	Oleuropein	5.95 ± 0.04
**22.6**	Luteolin glucoside	2.29 ± 0.02 ^d^
**23.1**	Oleuropein diglucoside	0.3785 ± 0.0001 ^b^
**23.6**	Oleuropein isomer 1	1.562 ± 0.007 ^b^
**24.5**	Ligstroside isomer 1	<LOQ
**24.7**	Oleuropein isomer 2	3.27 ± 0.01 ^b^
**26.2**	Ligstroside isomer 2	<LOQ
**26.9**	Luteolin	<LOQ

^a^ mg HyE/g dry extract; ^b^ mg OE/g dry extract; ^c^ mg LgE/g dry extract; ^d^ mg LuGE/g dry extract.

**Table 8:** In vitro antioxidant activity by DPPH of optimal extracts.

Olive by-Product Optimal Extract	DPPH (mg Trolox Equivalents/g Dry Extract)
**Olive Pomace**	128 ± 1
**Olive Leaves**	147 ± 1

## Data Availability

All of the data generated by this research have been included in the article. For any assistance, it is possible to contact the corresponding authors.

## Supplementary Materials

The following supporting information can be downloaded at: https://www.mdpi.com/article/10.3390/antiox14080938/s1, Table S1: Calibration equations of standards used to quantify olive by-products extracts.

## Abbreviations

The following abbreviations are used in this manuscript:

UAE	Ultrasound-Assisted Extraction
GRAS	Generally Recognized As Safe
OL	Olive Leaves
OP	Olive Pomace
TPC	Total Phenolic Compounds
HPLC-ESI-QTOF-MS	High Pressure Liquid Cromatography coupled to electrospray ionization quadrupole time-of-flight mass spectrometry
DPPH	2,2-Diphenyl-1-picrylhydrazyl
