# Supplementary material for: Optimized Ultrasound-Assisted Extraction for Enhanced Recovery of Valuable Phenolic Compounds from Olive By-Products
Source: Antioxidants (Basel). 2025 Jul 30;14(8):938. doi: 10.3390/antiox14080938 (PMC12382780; doi:10.3390/antiox14080938)
Supplement: Supplementary file 1 [file antioxidants-14-00938-s001.zip › antioxidants-3750935-supplementary.pdf]

Table S1. Calibration equations of standards used to quantify olive by-products extracts.

Calibration standard	Calibration equation	R^2 (%)	LOD (ppm)	LOQ (ppm)
**Apigenin**	$y = -199.57x^2 + 34920.38x + 455189.71$	98.81	0.240±0.005	0.80±0.02
**Hydroxytyrosol**	$y = -106.13x^2 + 39592.22x – 28192.98$	99.73	0.0267±0.0001	0.0891±0.0002
**Oleuropein**	$y = -175.54x^2 + 30419.50x + 89858.60$	98.72	0.169±0.005	0.56±0.01
**Verbascoside**	$y = -75.07x^2 + 14163.75x – 6062.83$	99.84	0.62±0.03	2.07±0.09
**Luteolin-7-*O*-glucoside**	$y = -179.33x^2 + 20074.41x + 69560.66$	98.52	0.03±0.01	1.00±0.05
**Coumaric acid**	$y = 2373.89x^2 - 6712.55x + 12846.62$	98.30	0.481±0.004	1.60±0.01
**Loganin**	$y = -154.82x^2 + 27558.71x + 120789.80$	98.07	0.055±0.003	0.18±0.01
